# Expression of NFIL3 and CEBPA regulated by IFNT induced-PGE2 in bovine endometrial stromal cells during the pre-implantation period

**DOI:** 10.3389/fendo.2023.1075030

**Published:** 2023-02-21

**Authors:** Rulan Bai, Kazuya Kusama, Yuta Matsuno, Hanako Bai, ﻿Toshihiro Sakurai, Koji Kimura, Kazuhiko Imakawa

**Affiliations:** ^1^National Key Laboratory of Veterinary Public Health Security, College of Veterinary Medicine, China Agricultural University, Beijing, China; ^2^Department of Endocrine Pharmacology, Tokyo University of Pharmacy and Life Sciences, Tokyo, Japan; ^3^Department of Genetics, Albert Einstein College of Medicine, Bronx, NY, United States; ^4^Laboratory of Animal Breeding and Reproduction, Research Faculty of Agriculture, Hokkaido University, Hokkaido, Japan; ^5^School of Pharmaceutical Science, Ohu University, Fukushima, Japan; ^6^Graduate School of ﻿Environmental and Life Science, Okayama University, Okayama, Japan; ^7^Research Institute of Agriculture, Tokai University, Kumamoto, Japan

**Keywords:** bovine, endometrium, stomal cell, prostaglandin E2, cyclic AMP

## Abstract

Prostaglandin E2 (PGE2) is considered as a luteoprotective factor, influencing the corpus luteum during the early pregnant period in the bovine species. Cyclic AMP (cAMP) is activated in response to PGE2 and plays a role in many physiological processes. The maternal recognition signal, interferon τ (IFNT), induces PGE2 secretion from the endometrial epithelial cells, the function of which in stroma cells has not been completely understood. In this study, PGE2 was found to activate cAMP in the bovine endometrial stromal cells (STRs). STRs were then treated with forskolin to activate the cAMP signaling, from which RNA extracted was subjected to global expression analysis. Transcripts related to transcription regulatory region nucleic acid binding of molecular function, nucleus of cellular component, and mitotic spindle organization of biological processes were up-regulated in cAMP-activated bovine STRs. An increase in the transcription factors, *NFIL3, CEBPA*, and *HIF1A via* the cAMP/PKA/CREB signaling pathway in the bovine STRs was also found by qPCR. Knockdown of *NFIL3*, *CEBPA*, or *HIF1A* blocked forskolin-induced *PTGS1/2* and *IGFBP1/3* expression. Moreover, NFIL3 and CEBPA were localized in endometrial stroma on pregnant day 17 (day 0 = estrous cycle), but not on cyclic day 17. These observations indicated that uterine PGE2 induced by conceptus IFNT is involved in the early pregnancy-related gene expression in endometrial stromal cells, which could facilitate pregnancy establishment in the bovine.

## Introduction

Prostaglandins (PGs) are essential for reproductive processes such as ovulation, fertilization, and embryo implantation in autocrine, paracrine, or occasionally endocrine fashion (1, 2). The corpus luteum (CL) is formed after ovulation and secretes progesterone to maintain pregnancy. The luteolytic function of PGF2α, produced by the endometrium, has been verified in humans and other mammals, whereas endometrial PGE2 has a luteoprotective effect during early pregnancy. Luteoprotective factor PGE2 is produced by bovine endometrial epithelial and stromal cells (EECs, STRs) (3, 4). PGE2 directly acts on luteal cells through the luteinizing hormone receptor, which induces progesterone synthesis. Bovine embryonic interferon τ (IFNT; a pregnancy recognition cytokine) starts to increase on day 7 of pregnancy (day 0 = day of estrus) and peaks on days 19-20, just after the initiation of conceptus attachment to the uterine epithelium, followed by a rapid IFNT decrease (5–8). IFNT is secreted by mononuclear trophoblast cells and acts as the maternal recognition signal in the ruminants (9, 10). IFNT downregulates the expression of endometrial oxytocin receptors and then maintains the corpus luteum function *via* inhibition of the luteolytic pulse of endometrial PGF2α (11–15). Therefore, the increase in the ratio of PGE2/PGF2α synthesis in the endometrium induced by IFNT is essential for maintaining the CL function during the early stage of pregnancy in ruminants.

PGE2 binds to the type E prostanoid (EP) receptors containing four subtypes of G-protein-coupled receptors EP1, EP2, EP3, and EP4. PGE2 plays distinct functions in various physiological processes caused by the differences in cellular distribution and downstream signaling pathways of each EP receptor. The highly expressed EP1 in the uterus promotes cell proliferation by activating PLC/PKC signaling, and its expression decreases sharply in the luteal phase (16). EP1 can work with EP3 inhibitory G protein to inhibit the cAMP pathway (16). Although *EP1* or *EP3*-deficient mice do not show abnormal pregnancy, *EP2*-null mice exhibit anovulation and embryo implantation dysfunction (17). Activation of EP2 and EP4 stimulates the cAMP/protein kinase A (PKA) signaling pathway, cAMP-response element binding protein (CREB), and MAPK signaling, ERK1/2 extracellular signal-regulated kinase-1/2 (ERK1/2). Several studies have also shown that EP2 and EP4 can enhance PI3K/Akt signaling (18). PGE2 stimulates the synthesis and secretion of PGE2 to form a positive feedback loop in endometrium and increases VEGFA to induce endometrial angiogenesis (19). Furthermore, PGE2 induces the synthesis and secretion of estrogen, catenin, and intercellular adhesion factors in porcine conceptuses (20).

During the peri-implantation period in ruminants, the trophoblast, CL, and the endometrium synthesize and secrete PGE2 (15, 21, 22). Intrauterine injection of PG synthase (PTGS) inhibitors during early pregnancy could suppress the conceptus elongation in the ovine and reduce the pregnancy rate in the bovine (3). PGs derived from the uterus, rather than the embryo, play roles in embryo development and implantation (23). IFNT-induced PGE2 prevents luteolysis (15), and the PGE2/EP2 signaling pathway is essential for endometrial maturation for receptivity to the embryo (24). The transcripts and protein levels of EP2 are found in the endometrium during the estrous cycle and increase in the bovine endometrial stroma on day 18 of pregnancy (25). IFNT is observed to up-regulate PGE2 in cultured endometrial epithelial cells and stromal cells (26–30). Moreover, the signaling pathway downstream of EP2 in bovine endometrial stromal cells at the peri-implantation stage is not fully understood. However, the physiological significance of the high expression of EP2 in bovine endometrial stromal cells at the peri-implantation stage is not fully understood. We, therefore, hypothesized that IFNT-induced PGE2 in bovine endometrial stromal cells (STRs) may affect the receptivity of conceptus *via* EP2/cAMP signaling at the pre-implantation stage. To investigate the physiological role of PGE2/cAMP in STRs, we extracted RNA from STRs, which had been treated with an adenylate cyclase activator, were subjected to RNA-sequence analysis.

## Materials and methods

### Cell preparation and culture conditions

Isolation and culture of endometrial cells were carried out as previously described (31–35). In brief, the uteri of healthy Holstein cows were obtained from a local abattoir in accordance with protocols approved by the local Institutional Animal Care, Use and Ethics Committee at Okayama University, Okayama, Japan. Uteri of the early luteal phase (days 2-5) were excised and immediately transported to the laboratory. The uterine lumen was trypsinized (0.3% w/v), from which endometrial epithelial cells (EECs) were isolated. After collection of the epithelial cells, the uterine lumen was washed and cut transversely. Intercaruncular endometrial strips were dissected from the myometrial layer with a scalpel and washed once. The endometrial strips were minced into small pieces and then digested with 0.05% collagenase I. After stirring for 60 min, endometrial stromal cells (STRs) were dissociated, filtered, and washed. Endometrial cells were then cultured on collagen type IA-coated plates in Dulbecco modified Eagle medium/F12 (DMEM/F12) (1:1) medium (Wako Pure Chemical Industries, Osaka, Japan) supplemented with 10% (v/v) newborn calf serum (Thermo Fisher Scientific), 2 mM glutamine (Thermo Fisher Scientific), and antibiotic/antimycotic solution (Thermo Fisher Scientific) at 37°C under 5% CO_2_ in humidified air. EECs or STRs placed onto collagen type IA-coated 12-well plate were further incubated with or without IFNT (1 µg/ml), FSK (50 µM), PGE2 (30 µM), EPAC-selective agonist (8-[4-chlorophenyltio]-2’-O-methyl cAMP; 500 µM), or PKA-selective agonist (N^6^-phenyl-cAMP; 500 µM) for 48 h.

### RNA extraction and quantitative RT-PCR

Using the ISOGEN reagent (Nippon Gene, Tokyo, Japan), total RNAs were extracted from cultured EECs or STRs according to the manufacturer’s protocols. The isolated RNA was reverse-transcribed to cDNA using ReverTra Ace qPCR RT Kit (Toyobo, Osaka, Japan), which was then subjected to qPCR amplification using PowerUP SYBR Green Master Mix (Thermo Fisher Scientific). All primers are listed in **Table 1**. The qPCR amplification was carried out on an Applied Biosystems STEP One Plus real-time PCR System (Applied Biosystems, Foster City, CA, USA). Amplification efficiencies of each target genes and the reference genes, glyceraldehyde-3-phosphate dehydrogenase (*GAPDH*) and actin beta (*ACTB*) were examined through their calibration curves and found to be comparable. Average threshold (Ct) values for each target were determined by Sequence Detection System software v2.3 (Applied Biosystems) (36).

**Table 1:** Primers for real-time qPCR analyses.

Name (Accession No.)	Sequence (5’—3’)	Product length (bp)
*GAPDH* NM_001034034	GCATCCCTGAGACAAGATGGTG	113
	CATTGATGGCAACGATGTCCAC	
*PTGS1* NM_001105323.1	ATGAGCCGGCAGGGTATCT	115
	AGTAACAGCAGGGGTTCACTG	
*PTGS2* NM_174445.2	CTCAGCGGTGCAGCAAATC	104
	CCTGTTCGGGTACAGTCACA	
*IGFBP1* NM_174554.3	TGCTGGACAGATTAGCCAGG	112
	GACGTCTCACACTGTTTGCTG	
*IGFBP3*	GCTACAAGCGTTGTTGGACG	107
NM_174556.1	CCGACTCACTGCCATTTCCT	
*HIF1A*	TGCTCATCAGTTGCCACTCC	117
NM_174339.3	TCCAAATCACCAGCATCCAGA	
*CEBPA*	TCGACATCAGCGCCTACATC	134
NM_176784.2	GTAGTCAAAGTCGTTGCCGC	
*NFIL3*	GAGCGCCTTTGTGGATGAGC	134
NM_001075240.1	CAGGGCCCTCCTGTGAATGT	
*VEGFA*	CAAACCTCACCAAAGCCAGC	107
NM_174216.2	GCCCACAGGGATTTTCTTGC	

### PGE2 ELISA

Endometrial cells were treated with recombinant IFNT (1 µg/ml) for 24 h. The culture medium was centrifuged at 10,000 g for 10 min at 4°C, and the concentration of PGE2 in the supernatant was determined using a sandwich ELISA Kit (Prostaglandin E2 ELISA Kit, abcam, Tokyo, Japan) according to the manufacturer’s instructions (37).

### Cyclic AMP assay

Total cAMP levels in endometrial cells, treated with IFNT, forskolin or PGE2 for 48 h, were determined using a competitive EIA kit (Cyclic AMP EIA kit, Cayman Chemical Company) according to the manufacturer’s recommendations. Briefly, cells were lysed for 10 min in 80 µl of 0.1 M HCl and were centrifuged at 1,000 x g at 4°C. The supernatant was used for the cAMP measurement (38).

### RNA sequencing and their gene ontology and pathway analyses

Total RNA for RNA-seq analysis was extracted from cultured EECs or STRs using Isogen (Nippon gene) according to the manufacturer’s instructions. High-throughput sequencing libraries were prepared using the TruSeq Stranded mRNA LT Sample Prep Kit (Illumina, San Diego, CA, USA) according to the manufacturer’s instructions, and the analysis was performed by Macrogen Japan (Kyoto, Japan). Primary sequencing data were deposited to the DNA Data Bank of Japan (DDBJ) Sequence Read Archive (https://www.ddbj.nig.ac.jp/dra/index-e.html) (accession numbers DRR413427 to DRR413432). Data analysis was performed as described previously (39). Briefly, trimmed sequences were analyzed on the basis of the TopHat/Cufflinks pipeline based on the bovine genome (bosTau8) and reference annotations obtained from UCSC genome browser (https://genome.ucsc.edu). Differential and significant gene expression analysis was performed with the use of fragments per kilo-base of gene locus summarized mRNA per million reads (FPKM). Genes were selected with the criteria of an absolute expression level >1 FPKM. The gene ontology (GO) and enriched signaling pathway analyses were performed with the Enrichr tool (http://amp.pharm.mssm.edu/Enrichr/ ).

### Western blot analysis

Cell lysates from the STR cultures were separated through SDS-PAGE and were then transferred onto polyvinylidene difluoride (PVDF) membranes (Bio-Rad, Hercules, CA, USA). After blocking with Block Ace reagent (DS Pharma Biomedical, Osaka, Japan), membranes were incubated with anti-CREB (1:2000, ab32515, abcam), phosphorylated (p-) CREB (1:2000, ab32096, abcam), p-ERK1/2 (1:2000, #4370, Cell signaling technology, Tokyo, Japan), p-p38MAPK (1:2000, #4511, Cell signaling technology), p-JNK (1:2000, #9251, Cell signaling technology), or ACTB (1:5000, ab1801, abcam) antibody. Immunoreactive bands were detected using enhanced chemiluminescence (EMD Millipore, Temecula, CA, USA) after incubation with horseradish peroxidase labeled anti-mouse, rabbit, or goat IgG (1:5000, Vector Laboratories, Burlingame, CA, USA). Signals were detected using C-DiGit Blot Scanner (LI-COR) and then band density was assessed with Image Studio DiGit software (version 5.2) (36).

### Transfection of small interfering RNA

STR cells were transfected with either a non-targeting control, or with nuclear factor interleukin 3 (*NFIL3*) (Sigma-Aldrich), CCAAT-enhancer-binding proteins alpha (*CEBPA*) (Sigma-Aldrich), or hypoxia-inducible factor (*HIF1A*) siRNA (Sigma-Aldrich) using Lipofectamine RNAiMAX (Thermo Fisher Scientific) (40).

### Immunohistochemistry

All animal procedures including uterine collections were performed in accordance with the guidelines of the Committee for Experimental Animals at Zennoh Embryo Transfer (ET) Center (Hokkaido, Japan) and the approval was also obtained from the Ethics Committee of the University of Tokyo (IRB number 7A-6-605). Paraffin sections of endometrial tissues were immunostained using antibodies targeting NFIL3, CEBPA and HIF1A, according to a previously described protocol (41). Briefly, the paraffin sections were rehydrated, boiled for 20 min in 10 mM citrate buffer (pH 6.0), and then incubated with an antibody against NFIL3 (1:100, Sigma-Aldrich, Saint Louis, MO, USA), CEBPA (1:100, ab15047, abcam), or HIF1A (1:100, ab463, abcam) overnight at 4°C. Subsequently, the paraffin sections were incubated with goat anti-rabbit IgG biotin conjugate (1:800 dilution, B8809, Sigma-Aldrich). The immunoreactivity was visualized by means of avidin-peroxidase (Sigma-Aldrich) and AEC substrate kit (Invitrogen) according to the manufacturer’s instructions.

### Statistical analysis

All experimental data from qPCR analyses represent the results obtained from three or more independent experiments each with triplicate assays. Data were expressed as the mean ± SEM. Significance was assessed using the Dunnett’s test. A P-value < 0.05 was considered statistically significant. In RNA-seq analysis, a false discovery rate-adjusted *P*-value (*q*-value) <0.05 was considered to represent statistical significance.

## Results

### 2 IFNT induces PGEin bovine EECs

**Figure 1:**
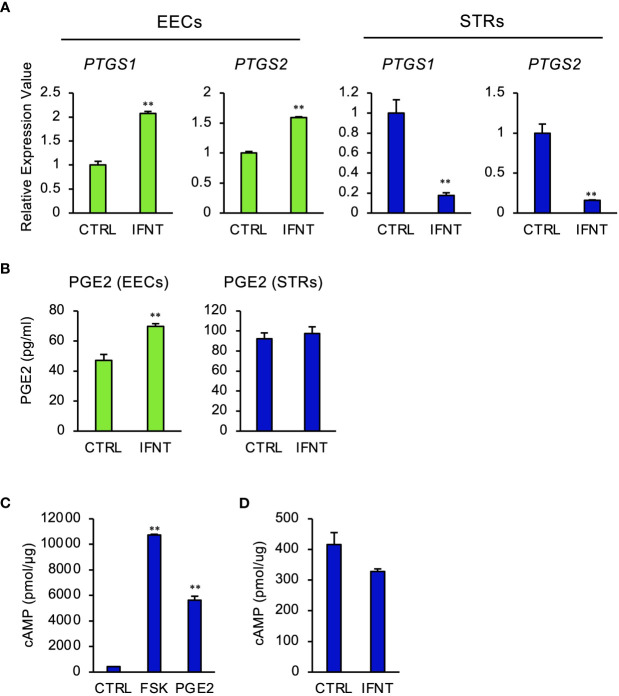
Regulation of PGE2 by IFNT in bovine endometrial cells. Bovine endometrial epithelial cells (EECs) or stromal cells (STRs) were treated with IFNT (1 µg/ml), FSK (50 µM), or PGE2 (30 µM) for 48 (h) **(A)** Transcript levels of prostaglandin synthase, PTGS1 and PTGS2, were measured by qPCR (n=3). *GAPDH* mRNA was used as the reference gene. Values represent the mean ±SEM of three independent experiments. **P<0.01. **(B)** PGE2 production in the culture media of the EECs or STRs was measured by the ELISA. Values represent the mean ±SEM of three independent experiments. **P<0.01. **(C, D)** The cAMP levels in the cell lysates were determined by EIA. The amount of cAMP was normalized to the amount of total cellular protein. The data from three independent experiments are presented. **p<0.01 vs. CTRL.

To investigate whether IFNT induced PGE2 in endometrial cells, EECs or STRs, were treated with IFNT. IFNT upregulated the expression of prostaglandin-endoperoxide synthase, PTGS1 and PTGS2 in EECs (**Figure 1A**), but those expressions were downregulated in STRs (**Figure 1B**). Secreted PGE2 in the culture media also increased when treated with IFNT in EECs, but not in STRs (**Figure 1C**). We next examined whether PGE2 stimulated STRs to generate intracellular cAMP. PGE2 increased intracellular cAMP level as well as FSK, an activator of adenylate cyclase (**Figure 1D**). However, IFNT did not stimulate the generation of cAMP in STRs (**Figure 1E**).

### Cyclic AMP upregulates the expression of transcription factors and pregnancy-related genes in STRs

**Figure 2:**
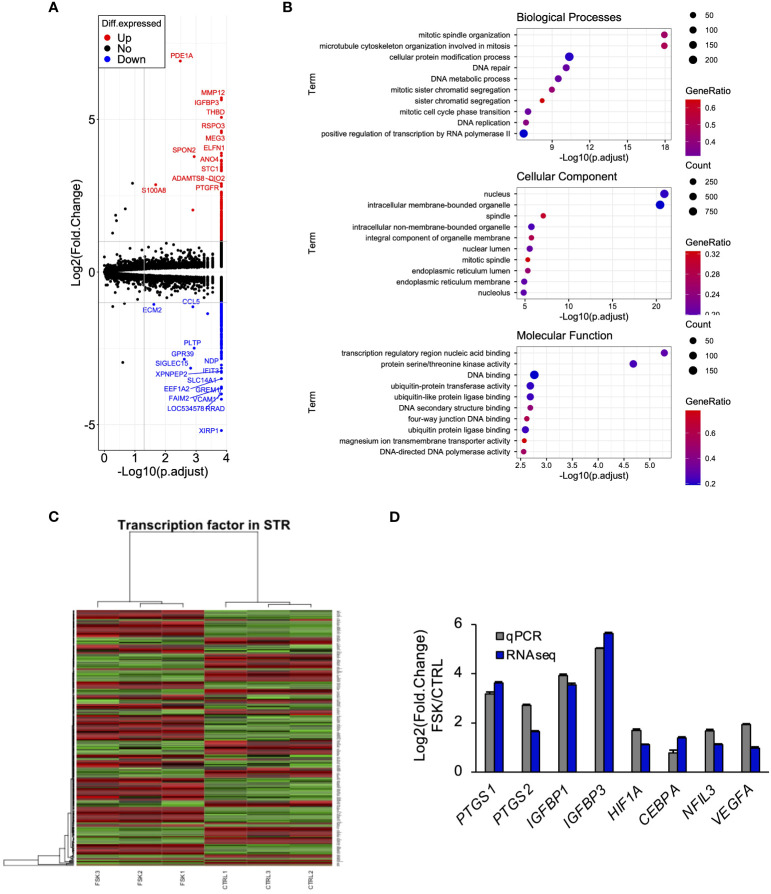
Cyclic AMP upregulates the expression of transcription factors and pregnancy-related genes in STRs. STRs were treated with FSK (50 µM) for 48 (h) Next, RNA was extracted and subjected to RNA sequencing (RNA-Seq; **(A–C)** or qPCR **(D)**. **(A)** Volcano plot showing expression of transcripts identified by RNA-seq. The transcripts highlighted in red or blue were differentially expressed by 2-fold (*P* < 0.05). **(B)** Differentially expressed genes were functionally classified using Gene Ontology analysis with the biological process, cellular component, or molecular function data sets. **(C)** Heat-map study of transcription factors in CTRL and FSK-treated STRs. Transcripts upregulated are shown in red, and those downregulated are shown in green. **(D)** Expression of *PTGS1/2*, *IGFBP1/3*, *NFIL3*, *CEBPA*, *HIF1A*, and *VEGF* genes in STRs was measured by qPCR (n=3). *GAPDH* mRNA was used as the reference gene. Values represent the mean ±SEM of three independent experiments.

To explore the effect of PGE2 on gene expression through cAMP signaling pathway in STRs, we performed RNA-seq analysis. This identified 552 DEGs, of which 244 were downregulated and 308 were upregulated (**Figure 2A**). GO enrichment analyses of upregulated genes identified those related to mitosis-, nucleus-, or transcriptional regulation-related terms (**Figure 2B**). GO analysis enabled us to suppose the involvement of transcription factors in STRs when stimulated with cAMP. The DEGs were further compared with the transcription factor database, from which a heat map was generated (**Figure 2C**). The results from the qPCR analysis also revealed an effect of an increase in cAMP on the expression of PGE2 synthase, pregnancy-related, and several transcription factors in STRs, which were similar to those found by RNA-seq analysis (**Figure 2D**).

### PGE2 affects gene expression *via* cAMP/PKA/CREB signaling pathway in STRs

**Figure 3:**
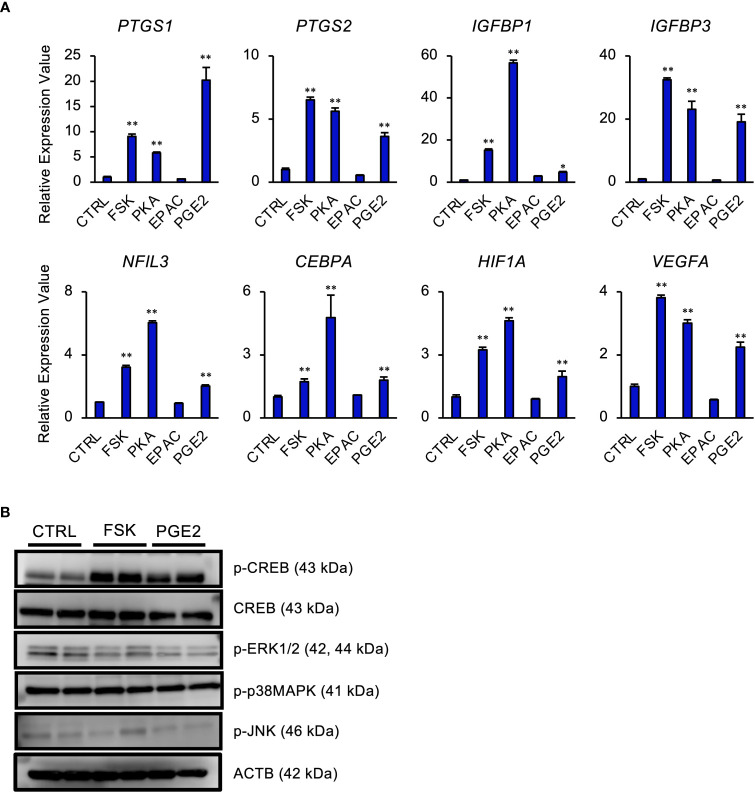
PGE2 affects gene expression *via* cAMP/PKA/CREB signaling pathway in STRs. STRs were treated with FSK (50 µM), PKA-selective agonist (500 µM), EPAC-selective agonist (500 µM), or PGE2 (30 µM) for 48 (h) **(A)** Expression of *PTGS1/2*, *IGFBP1/3*, *NFIL3*, *CEBPA*, *HIF1A*, and *VEGF* genes in STRs was measured by qPCR (n=3). *GAPDH* mRNA was used as the reference gene. Values represent the mean ±SEM of three independent experiments. *P<0.05, **P<0.01 *vs*. CTRL. **(B)** Lysates prepared from STRs that had been treated with FSK (50 µM) or PGE2 (30 µM) for 24 h were subjected to immunoblotting to determine phosphorylated (p-) CREB, p-ERK1/2, p-p38MAPK, and p-JNK protein levels. ACTB served as a loading control.

Intracellular cAMP activates two signaling pathways, PKA and exchange protein directly activated by cAMP (EPAC) pathways in STRs (42). To determine the effect of PGE2/cAMP on gene expression, STRs were treated with PKA- or EPAC-selective agonist. PKA-selective agonist upregulated the expression of *PTGS1/2*, IGF binding proteins (*IGFBP*) *1/3*, *NFIL3*, *CEBPA*, *HIF1A*, and vascular endothelial growth factor (*VEGF*) as well as FSK and PGE2 treatment, however, they were unaffected by the EPAC-selective agonist (**Figure 3A**). Further investigation of which intracellular signaling pathways were activated by PGE2/cAMP revealed that PGE2 increased phosphorylated CREB and decreased phosphorylated ERK1/2, but did not alter the activation of other p38MAPK and JNK in STRs (**Figure 3B**).

### NFIL3, CEBPA, or HIF1A mediates cAMP signaling pathway in STRs

**Figure 4:**
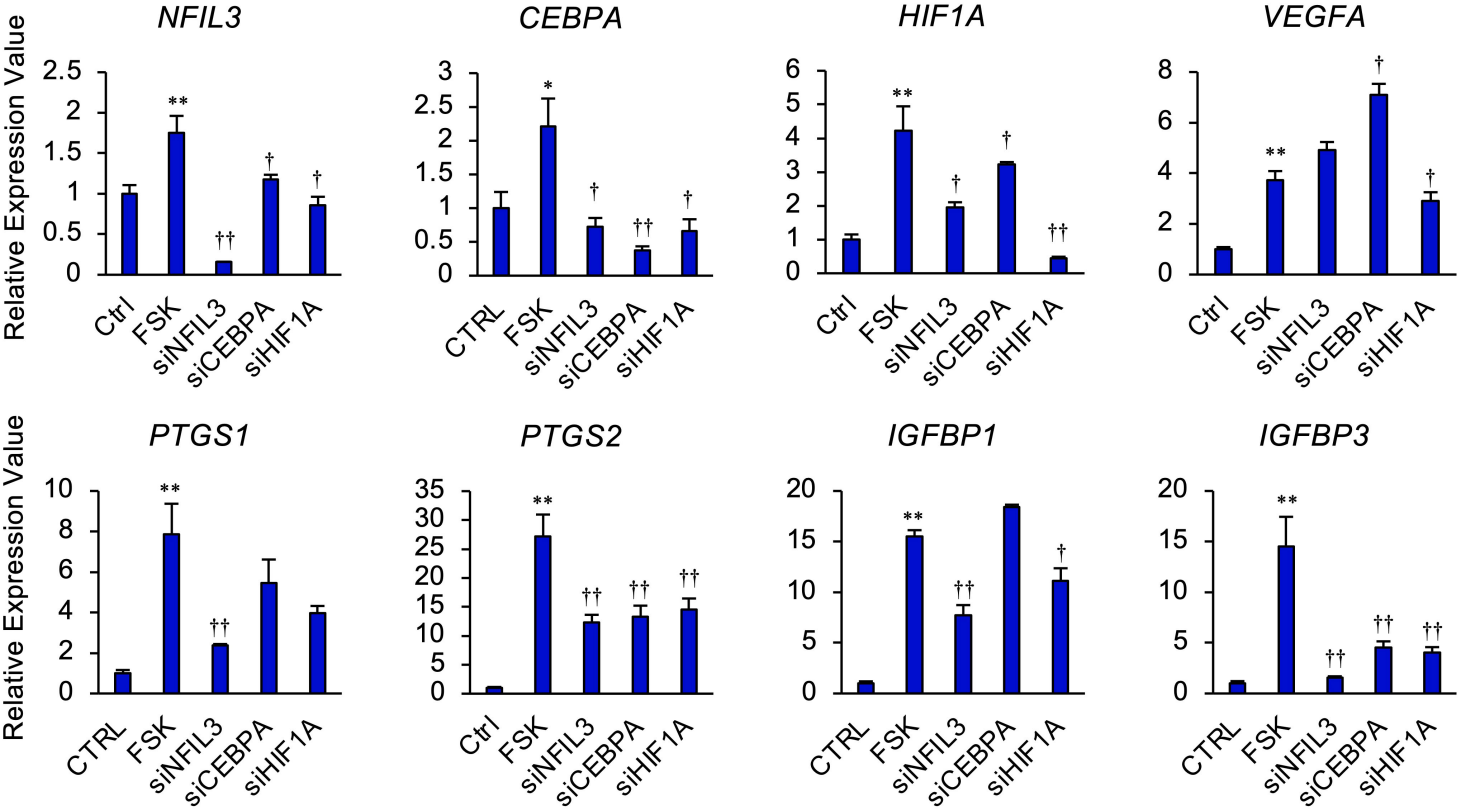
NFIL3, CEBPA, or HIF1A mediates cAMP signaling pathway in STRs. STRs transfected with *NFIL3*, *CEBPA*, or *HIF1A* siRNA were treated with FSK (50 µM) for 48 h. Expression of *PTGS1/2*, *IGFBP1/3*, *NFIL3*, *CEBPA*, *HIF1A*, and *VEGF* genes in STRs was measured by qPCR (n=3). *GAPDH* mRNA was used as the reference gene. Values represent the mean ±SEM of three independent experiments. *P<0.05, **P<0.01 *vs*. CTRL, ^†^*P*<0.05, ^††^*P*<0.01 *vs*. FSK.

We next investigated whether NFIL3, CEBPA, or HIF1A significantly affected the expression of *PTGS1/2*, *IGFBP1/3*, and *VEGF* in STRs (**Figure 4**). In the presence of FSK, *NFIL3* knockdown reduced the expression of *PTGS1/2* and *IGFBP1/3*. *CEBPA* knockdown also reduced the expression of *PTGS2* and *IGFBP3*. Furthermore, *HIF1A* knockdown reduced the expression of *VEGFA*, *PTGS2*, and *IGFBP1/3*.

### NFIL3, CEBPA, and HIF1A are localized in endometrial stroma and epithelium during the pre-implantation period

**Figure 5:**
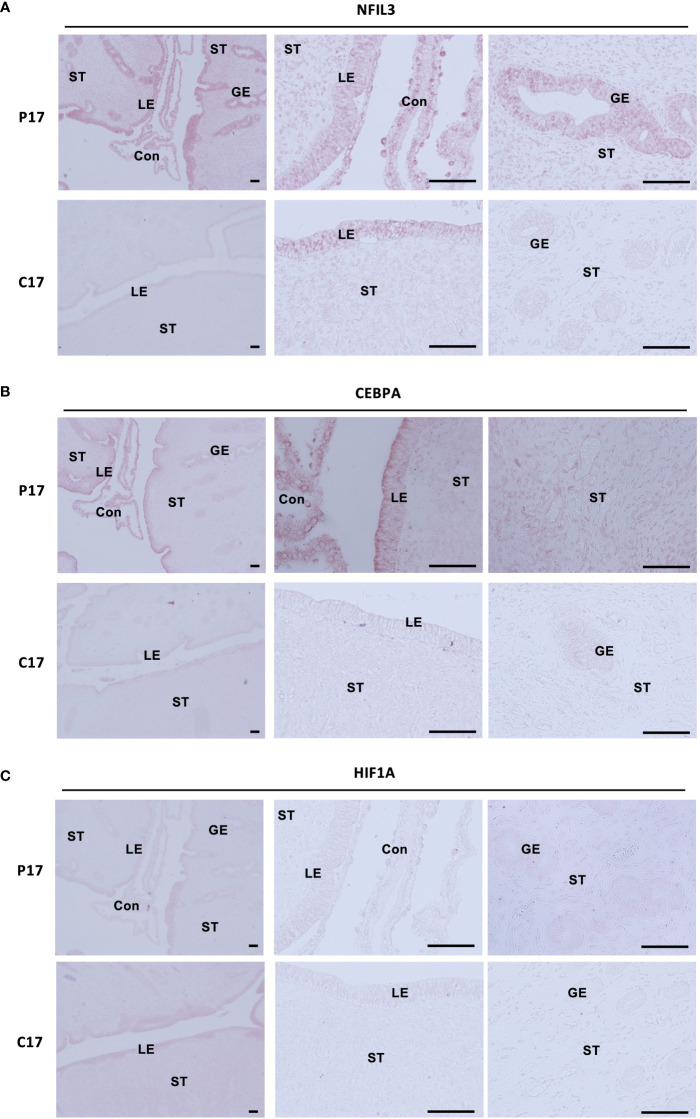
Localization of NFIL3, CEBPA, or HIF1A in the endometrial epithelium and stroma during pre-implantation periods. Sections of bovine endometrial tissues were immunostained for NFIL3 **(A)**, CEBPA **(B)**, or HIF1A **(C)**. P17, pregnant day 17; C17, cyclic day 17; ST, stroma; GE, glandular epithelium; LE, luminal epithelium; Con, conceptus. Scale bar = 100 μm.

Expression of NFIL3, CEBPA, and HIF1A in endometrial tissues on day 17, on which IFNT was maximally secreted from conceptuses, was examined by immunohistochemistry (**Figures 5A–C**). NFIL3, CEBPA, and HIF1A were expressed in stroma and luminal and glandular epithelium of pregnant animals, whereas NFIL3, CEBPA, and HIF1A were slightly expressed in only epithelium on cyclic day 17 in the absence of conceptus in the uterus.

## Discussion

**Figure 6:**
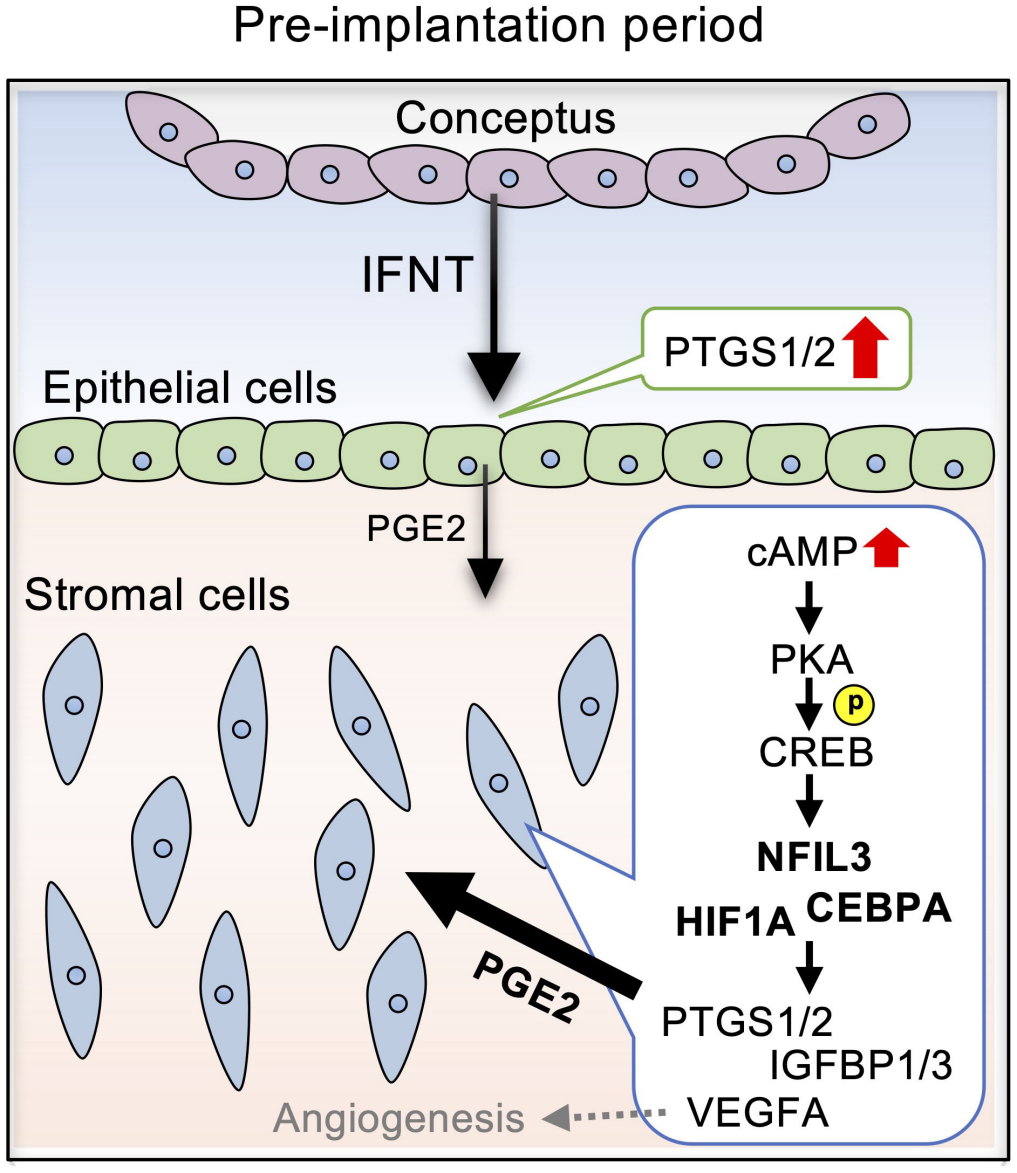
Diagram illustrating the effects of PGE2 on the expression of pregnancy-related factors *via* NFIL3, CEBPA, and HIF1A in bovine endometrial stromal cells. IFNT secreted from conceptuses acts on the endometrial epithelial cells, stimulating PGE2 production. PGE2 secreted from the epithelial cells activates cAMP signaling in the stromal cells, which is mediated by PKA/CREB. The cAMP signaling pathway upregulates the expression NFIL3, CEBPA, and HIF1A, which regulates PTGS1/2, IGFBP1/3, and VEGF.

In this study, we demonstrated that PGE2 upregulated the pregnancy-associated transcription factors, NFIL3, CEBPA, and HIF1A, *via* the cAMP/PKA/CREB signaling pathway in bovine endometrial stroma (**Figure 6**). Upregulation of these transcription factors in stromal cells was further confirmed by the observations of their localization in the uterine tissue of day 17 pregnant cows as compared to those in day 17 cyclic cows. These results provided evidence that PGE2 regulated the expression of pregnancy-associated transcription factors in bovine endometrial stroma cells.

In the present study, IFNT increased the secretion of PGE2 and the expression of PGs synthase *PTGS1* and *PTGS2* (known as cyclooxygenase 1 and 2, respectively) in EECs, but not in STRs (**Figure 1**). It was found that the IFNT receptors, IFNAR1 and IFNAR2, shared by all of the type I IFNs, are expressed in the endometrial epithelium, and that these expressions are much higher than those in the stroma in ruminants. These results suggest that the epithelium may be more responsive to IFNT than the stroma (43–45). As PGE2 is generally known as an autocrine and paracrine factor (2) and has an ability to increase intercellular adhesion factors (20), these results indicated that PGE2 secretion from endometrial epithelial cells stimulated by IFNT might regulate the epithelial and stromal cells to facilitate conceptus adhesion to the endometrium at the early stage of pregnancy in the bovine.

The studies presented here also demonstrated that when the endometrial stroma cells were treated with PGE2, the production of cAMP increased over 10 times (**Figure 1**), which is consistent with the results of others (46), whereas cAMP level was not altered by the IFNT treatment. EP2 is one subtype of four PGE2 receptors and is the only one identified as critical for ovulation and embryo implantation in mice (47). The expression of EP2 in the bovine increases on day 18 of pregnancy primarily in the endometrial stroma, when the conceptus begins to attach to the uterus (10, 25). These results and ours suggest that the production of PGE2 from endometrial epithelium might activate the cAMP signaling pathway through binding with the receptor EP2 in bovine stromal cells.

According to the functional GO analysis, the two most notable upregulation of biological processes in bovine endometrial stromal cells induced by cAMP were of the mitotic spindle organization and microtubule cytoskeleton organization involved in mitosis. Additionally, the nucleus and intracellular membrane-bounded organelle in the cellular component also increased significantly (**Figure 2B**). In ruminants, although decidualization in the endometrium does not occur, bovine endometrium is also remodeled in each estrous cycle, including cell proliferation in stroma cells (48). Our data indicated that the effect on the proliferation within the endometrium may partly be regulated by PGE2/cAMP and steroid hormones. More importantly, the upregulated genes induced by cAMP were shown to be most associated with transcription regulatory region nucleic acid binding in molecular function. Previously, we found that an increase in intracellular cAMP is subsequently involved in the activation of PKA and EPAC in endometrial stromal cells (42). The cAMP has a different affinity for EPAC and PKA; furthermore, different signaling pathways conduct multiple beneficial and deleterious actions (49). In this study, the cAMP/PKA/CREB pathway played an important role in the regulation of endometrial stromal cell gene expression (**Figure 3**).

NFIL3, also known as adenovirus E4 promoter-binding protein 4 (E4BP4), is a ubiquitously expressed basic leucine zipper transcription factor and is reported as a master regulator of NK cell differentiation, ovulation, placentation, and embryonic development (50–52). Our histochemical results showed that NFIL3 was localized in the bovine trophoblast on day 17 (**Figure 5A**). It is consistent with the findings that *Nfil3* is expressed by early trophoblasts in mice and that deficiency of trophoblastic *Nfil3* causes abnormal implantation and placentation (52). The results presented in this study identified that endometrial NFIL3 was highly expressed in the endometrial epithelium, stroma, and glandular epithelium in day 17 pregnant cattle, but was not detectable in cyclic day 17 uterus except in the luminal epithelium (**Figure 5**). Moreover, the knockdown of *NFIL3* could significantly block FSK-induced *PTGS1/2* and *IGFBP1/3*. CEBPA exhibits a similar expression pattern as NFIL3 in this study. NFIL3 binds to CEBPs including CEBPA upon a C/EBP (CCAAT/Enhancer Binding Protein) DNA binding motif (53). CEBPA is an important transcription factor associated with hematopoietic differentiation (54) and upon interaction with CDK2 and CDK4, CEBPA inhibits these kinases and leads cultured cells to stop dividing or induce cell growth and proliferation (55, 56). CEBPA may play role in the regulation of *PTGS1/2* and *IGFBP1/3* in cooperation with NFIL3 in endometrial stromal cells. IGFBP1/3 are found as specifically expressed in the endometrium, and perform the functions of cell proliferation, migration, and attachment in the ruminants. IGFBP3 is expressed in the stroma of bovine endometria, and IGFBP1 functions as an adhesion molecule bridge of trophoblast and endometrium (57, 58). PTGS2 is induced by EP2 activation in bovine endometrial epithelium *via* PKA/ERK pathways (59). Thus, stromal NFIL3 and CEBPA might regulate the gene expression involved in conceptus elongation and the tight adhesion between conceptus and uterus at the early pregnancy stage in the bovine. Moreover, *Nfil3*^-/-^ male*Nfil3*^-/-^ female mating results in low fecundity, and *Nfil3*^-/-^ females exhibit delayed uterine lumen closure and antimesometrial decidua differentiation in mice (52). Decidual NK cells have heterogeneity and are involved in neoangiogenesis and immune modulation during the first trimester of pregnancy (60). Reduction in decidual NK cell numbers is detected in *Nfil3*^-/-^ mouse. Considering that early pregnancy in the bovine is accompanied by a marked increase in the proportion of endometrial immune cells expressing markers for NK cells and cytotoxic T cells (61), stromal NFIL3 might play a role in maternal immune tolerance as well as endometrial cells proliferation. Interestingly, it is observed that the interface membrane separating maternal and fetal labyrinthine vessels is significantly thicker in *Nfil3*^-/-^ mouse, ﻿potentially affecting the nutrient and waste exchanges in mice placentas (52). As a previous report has demonstrated, the inhibition of VEGF induces thickening of the endothelium with less fenestration in choriocapillaris (62). In the bovine endometrial stromal cells, suppression of NFIL3 could not block the induction of VEGFA stimulated by FSK. Not only NFIL3 but the other two transcription factors chosen in the present study also could not inhibit the increasing expression of stromal VEGFA stimulated by cAMP. In contrast to the haemochorial placenta of rodents, the synepitheliochorial placenta of bovine resembles a polarised uterine epithelial barrier facing the fetal chorionic (trophoblast) epithelium, including caruncular, the part fuses with embryo cotyledons to form a placenta during pregnancy, and intercaruncular. It is observed that significantly higher expression of VEGFA at the preimplantation stage in the caruncular endometrium area forms part of the embryo-maternal interface (63). Taken together, these results indicate NFIL3 might play a different role in bovine endometrium compared to that in mice, and the regulatory mechanism of VEGFA in the bovine stroma still needs to be elucidated.

In summary, we propose that PGE2 enhances NFIL3 and CEBPA expression in endometrial stromal cells *via* the cAMP/PKA/CREB pathway, which may facilitate conceptus development, cell proliferation, and immune tolerance, resulting in the establishment of pregnancy at the early stage in the bovine.

## Data availability statement

The data presented in the study are deposited in the DNA Data Bank of Japan (DDBJ) Sequence Read Archive repository (https://www.ddbj.nig.ac.jp/dra/index-e.html), accession number DRR413427 to DRR413432.

## Ethics statement

The animal study was reviewed and approved by Ethics Committee at Okayama University.

## Author contributions

RB and KKu contributed to data acquisition. RB and KKu contributed to data analysis. YM, TS and HB prepared tissue sections. RB and KKi isolated primary endometrial stromal cells. RB, KKu and KI contributed to study conception, design, and manuscript preparation. All authors contributed to the article and approved the submitted version.
