# Supplementary material for: Expression of NFIL3 and CEBPA regulated by IFNT induced-PGE2 in bovine endometrial stromal cells during the pre-implantation period
Source: Front Endocrinol (Lausanne). 2023 Feb 21;14:1075030. doi: 10.3389/fendo.2023.1075030 (PMC10010167; doi:10.3389/fendo.2023.1075030)

Supplemantary information.

Full images of western blots shown in Figure 3 (B)

These pictures indicate the uncropped western blot data.

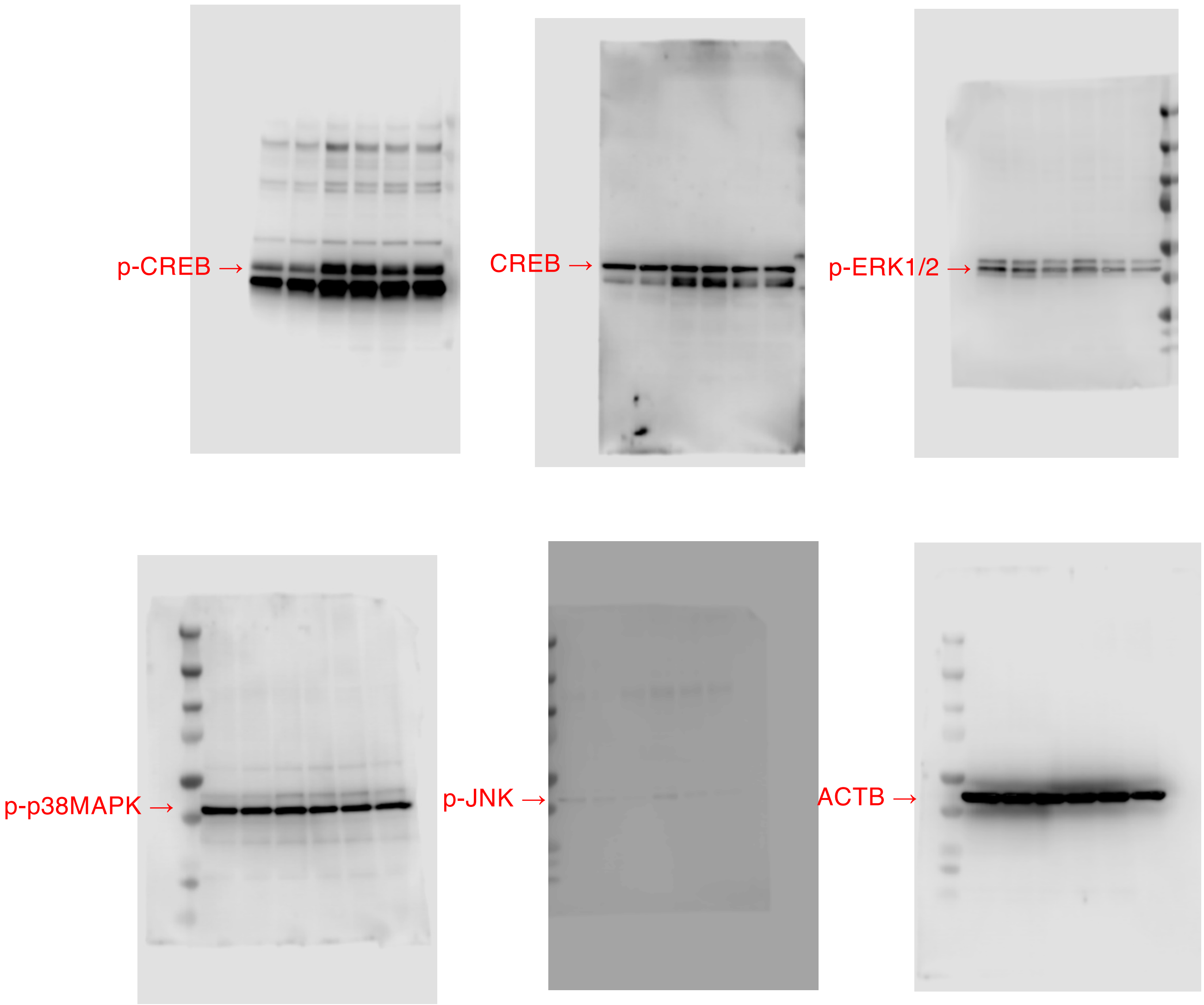

Supplement: Supplementary file 1 [file DataSheet_1.pdf]
